# The Psychosocial Aspects of Vegetarian Diets: A Cross-Sectional Study of the Motivations, Risks, and Limitations in Daily Life

**DOI:** 10.3390/nu16152504

**Published:** 2024-08-01

**Authors:** Agnieszka Białek-Dratwa, Wiktoria Stoń, Wiktoria Staśkiewicz-Bartecka, Mateusz Grajek, Karolina Krupa-Kotara, Oskar Kowalski

**Affiliations:** 1 Department of Human Nutrition, Department of Dietetics, Faculty of Public Health in Bytom, Medical University of Silesia in Katowice, ul. Jordana 19, 41-808 Zabrze, Poland; 2 Department of Food Technology and Quality Assessment, School of Public Health in Bytom, Medical University of Silesia in Katowice, ul. Jordana 19, 41-808 Zabrze, Poland; wstaskiewicz@sum.edu.pl; 3 Department of Public Health, School of Public Health in Bytom, Medical University of Silesia in Katowice, ul. Piekarska 18, 41-902 Bytom, Poland; 4 Department of Epidemiology, School of Public Health in Bytom, Medical University of Silesia in Katowice, ul. Piekarska 18, 41-902 Bytom, Poland

**Keywords:** vegetarian diet, vegan diet, orthorexia, eating disorders, psychosocial aspects

## Abstract

Introduction: The popularity of vegetarian and vegan diets is linked to various motivations, such as health, ethics, ecology, and social and religious influence. India has the highest proportion of vegetarians and vegans. The practise of these diets is linked to moral and health reasons and environmental concerns. Vegetarianism may also be associated with eating disorders such as orthorexia (ON). Aim: The main aim of this study was to determine the psychosocial aspects of vegetarian diets. Understanding these aspects is crucial for identifying potential risks and developing effective interventions. This study investigated the reasons for following vegetarian diets, the duration of dietary adherence, the occurrence of feelings of restriction in selected situations, and the risk of orthorexia and other eating disorders. Methods: A questionnaire survey was conducted among 186 individuals (82 vegetarians and 104 traditional dieters) between October 2023 and April 2024. The survey was administered via a Computer-Assisted Web Interview (CAWI) using Google Forms, distributed through social media, forums, and private messages. The inclusion criteria for the study group included consent, an age over 18, and a vegetarian diet, excluding those with eating disorders or diseases requiring strict diet therapy. The control group criteria were similar, excluding vegetarians and those requiring special diets. Four unreliable questionnaires were excluded from the analysis. The survey consisted of four sections: metric data, the ORTO-15 questionnaire, the EAT-26 questionnaire, and the TFEQ-13 questionnaire. Results: The main motivations for following vegetarian diets were ethical and environmental (86.9%) and health (32.1%) reasons. Over half of the vegetarians had been following a plant-based diet for over five years. Vegetarians were more likely to feel restricted in restaurants and when grocery shopping. The ORTO-15 results indicate a higher risk of orthorexia among vegetarians (48.8% vs. 29.4% in the control group; *p* = 0.00673). The EAT-26 questionnaire showed a higher, but not statistically significant, risk of eating disorders among vegetarians (23.8% vs. 14.7%; *p* = 0.11391). The TFEQ-13 showed no significant differences between groups (Subscale 1: food restriction, *p* = 0.77279; Subscale 2: lack of control in overeating, *p* = 0.91935; Subscale 3: eating under the influence of emotions, *p* = 0.16612). Conclusions: This study concluded that ethical and environmental considerations and a belief in health benefits mainly drive vegetarians. An analysis of BMI revealed no significant differences between groups. The ORTO-15 results suggest a higher risk of orthorexia among vegetarians. The EAT-26 indicated a higher, but not statistically significant, risk of eating disorders among vegetarians and vegans. The TFEQ-13 showed no significant differences in restrictive eating, lack of control in overeating, and emotional eating. Vegetarians were likelier to encounter dietary difficulties in restaurants and shopping but less likely to feel socially excluded.

## 1. Introduction

Currently, due to widespread access to the internet and increasing opportunities to expand knowledge, a steadily increasing number of people are choosing to change their lifestyles, including their diets [1]. In recent years, the vegetarian diet and its variations have gained popularity. There are many motives for following a plant-based diet. These include health aspects, morality, the impact on ecology and the environment, the society to which the person belongs, prevailing trends, and religion [1,2,3,4,5,6,7]. Studies indicate that ethical considerations and health concerns are the main reasons for choosing a vegetarian or vegan lifestyle [7,8,9,10,11,12,13].

Many vegetarians and vegans choose plant-based diets for moral reasons based on respect for living beings, compassion, and sustainability. They disapprove of animal suffering by avoiding animal products, such as leather and fur products, and using animals to test such products. Veganism and vegetarianism are value systems related to animal rights protection, ecology, and conscious eating [14,15,16]. An essential reason for implementing a vegetarian diet is concern for the environment and the need to influence the ecological situation. A meat-rich diet contributes to the waste of plants and water by mass animal farming, causing significant pollution and greenhouse gas emissions, accounting for about 30% of global emissions. Animal farming generates CO_2_ and methane and contributes to deforestation and biodiversity loss. Studies indicate that reducing meat consumption can mitigate climate change [14,17,18,19].

The practise of vegetarian diets can be explained by Maslow’s pyramid of needs in which the need to belong motivates the implementation of such a diet. This need includes maintaining contact and relationships based on acceptance, which increases the sense of security and reinforces the feeling of acceptance [20,21]. Vegetarianism is not only a diet but also a lifestyle and an expression of a worldview related to sustainability. The community of vegetarians and vegans manifests itself in forums and discussion groups where they share thoughts, recipes, and experiences. Participation in such groups satisfies the need to belong and facilitates relationships with like-minded people [22,23].

Vegetarian diets can prevent and treat diseases such as type 2 diabetes, hypertension, lipid disorders, and some cancers. It is often associated with a desire to maintain or reduce body weight. Studies show that vegetarians and vegans have a lower BMI than people on a traditional diet. Vegans reported the most significant weight loss (7.5 per cent after 6 months), and vegetarians lost an average of 2 kg more than individuals eating animal products [24,25].

Orthorexia (ON) is a relatively new eating disorder whose definition and diagnostic criteria are still under scientific debate. Currently, ON is not recognised as a psychopathological disorder in the DSM-5 or ICD-11 [26,27], and therefore, there are no established and validated diagnostic criteria. There is an ongoing debate in the literature as to whether ON should be considered a behavioural/lifestyle phenomenon or a psychiatric disorder. However, some studies describe specific clinical features of the disorder. The current definition of ON includes the obsession, fixation, and preoccupation with maintaining a healthy diet [28,29]. It is characterised by an obsession with eating only healthy foods and eliminating products that may negatively affect health. This attitude leads to significant dietary restrictions and even a fear of certain foods [30,31]. In people with ON, these eating behaviours become obsessive worries accompanied by pathological levels of worry and stress about food [32,33].

The development of ON begins with an innocent attempt to improve health through a healthy diet, but over time, the list of eliminated foods grows. These restrictions can lead to adverse health outcomes, such as an impaired quality of life, eating disorders, and malnutrition. Diet becomes central to life, leading to numerous dietary and social restrictions. People with orthorexia focus excessively on food, lose enjoyment of meals, develop cooking rituals, and feel the need to constantly control their eating, which can result in unintentional weight loss [30,34].

The characteristics of orthorexia include the elimination of processed and potentially harmful products from one’s diet, careful and time-consuming meal planning, meticulous label reading, and attention to the nutritional value of products. People with orthorexia spend much time reading about food and its potential harm and avoid social gatherings due to fear of consuming products of unknown origin [31].

Research suggests a link between vegetarian diets and eating disorders, as they can serve as a way to justify the avoidance of certain food groups and eating situations, masking restrictive eating patterns, and weight control. This finding raises concerns about the potential risks of vegetarian diets. Researchers have shown that women on vegetarian diets are more likely to exhibit disordered eating attitudes and behaviours than women on a traditional diet [2,34]. Furthermore, adherence to specific elimination diets (e.g., vegetarian, vegan, fruitarian, and vitarian diets) is associated with orthorexic eating patterns. Due to the ever-decreasing number of ‘allowed’ and ‘healthy’ foods, individuals who are omnivores may restrict their diets from traditional to vegetarian, and ultimately to vegan or one of the stricter varieties—fruitarian or vitarian [34].

A common feature of vegetarianism, its variants, and orthorexia is categorising food into ‘allowed’ and ‘not allowed’ items and the application of rigid dietary rules. Both approaches can lead to an excessive focus on food and a sense of exclusion in social situations, such as going out to restaurants with friends [35].

Despite some commonalities, it is not possible to conclude unequivocally that a vegetarian or vegan lifestyle is closely associated with orthorexia. The evidence that vegetarian diets are a risk factor for eating disorders is inconsistent. Some studies indicate that vegetarians and vegans are more likely to exhibit orthorexic behaviours than omnivores. In contrast, other studies show that people on vegetarian diets have less pathologically strict eating patterns than non-vegetarians [34].

The aim of this study was to analyse the psychosocial aspects of vegetarian diets compared to traditional diets. This study aimed to understand the impact of diet on the mental health, physical health, and daily lives of people following different dietary models by examining the reasons for following vegetarian diets, the duration of dietary adherence, the occurrence of feelings of restriction in selected situations, and the risk of orthorexia and other eating disorders. This study hypothesised that those following vegetarian diets would be more likely than those on traditional diets to cite ethical and environmental considerations as the main reasons for choosing their diets. There is a higher risk of orthorexia among those on vegetarian diets, as measured using the ORTO-15 questionnaire. Those on vegetarian diets will have a higher risk of eating disorders, as determined using the EAT-26 questionnaire. Vegetarians are more likely to feel restricted by their diets in their daily lives, including in restaurants, shopping, and social interactions.

## 2. Materials and Methods

### 2.1. Conducting the Survey

The survey was conducted between October 2023 and April 2024. The survey included 186 people aged eighteen or over. The survey group consisted of 82 people declaring that they follow a vegetarian diet or variations of it. In comparison, the control group consisted of 104 people who declared that they do not follow any vegetarian diet or exclude some animal products but do not consider that they follow any vegetarian diet. The survey was conducted with a Computer-Assisted Web Interview (CAWI) using Google Forms (https://www.google.pl/intl/pl/forms/about/, accessed on 14 July 2024). The questionnaire was shared on social media, forums, and online groups—mainly on the Facebook platform for vegetarians and vegans. The questionnaire for the control group, on the other hand, was distributed via individual private messages using a recruitment method based on the snowball effect of each participant being asked to pass the questionnaire on to further potential respondents.

The Declaration of Helsinki and the Act on the Profession of Physicians and Dentists conducted the study. The Bioethics Committee of the Medical University of Silesia in Katowice evaluated and approved the study protocol (PCN/0022/KB/68/I/20).

The inclusion criteria for the study group were consent to participate, the declaration of being a vegetarian or vegan, aged above 18 years, no diagnosed eating disorders, and no diseases requiring strict diet therapy, e.g., food allergy, renal failure, or gout. Inclusion criteria for the control group were consent to participate in the study, aged above 18 years, no declaration of being vegetarian or vegan, and no diseases requiring a special, very individual, or restrictive diet. The exclusion criteria for both the study and control groups were an incomplete questionnaire and a questionnaire completed unreliably. To verify the reliability of the completion of the questionnaire, 3 control questions placed in different parts of the questionnaire were used. Four unreliably completed questionnaires were excluded from the study.

### 2.2. Questionnaire Survey

The study of psychosocial aspects of vegetarian diets was carried out using a survey questionnaire, which included an author’s section on metric data and diet and a section containing three validated questionnaires: the ORTO-15, EAT-26, and TFEQ-13. The anonymity of those taking part voluntarily was fully preserved.

The survey questionnaire consisted of four sections. The first section contained six questions on metric data, such as gender, birth year, weight, height, work activity, and education. There were three closed-ended single-choice questions on gender (female, male, or other), work activity (unemployed, working, student, working student, or other), and education (primary, vocational, junior high, high school, or university) and three open-ended questions on age, weight, and height. The questions in this part of the questionnaire were ‘indicate your metric data’ and ‘enter your age/height/weight’. Body weight and height questions were used to calculate the body mass index. Values between 18.5 and 24.9 kg/m^2^ were taken as the average body mass index. Underweight was defined as a BMI below 18.5 kg/m^2^, while overweight was defined as a BMI between 25.0 and 29.9 kg/m^2^. For obesity, the body mass index was 30.0 kg/m^2^ or more [36].

The second section of the survey contained questions from the ORTO-15 questionnaire. The ORTO-15 questionnaire is one of the tools used to determine the presence of orthorexia in a respondent. It contains fifteen questions through which obsessive attitudes about eating (purchasing, preparing, and consuming healthy food) are assessed. We used the following questions in the questionnaire: When eating, do you pay attention to the calories of the food? When you go to a food store, do you feel confused? In the last 3 months, did the thought of food worry you? Are your eating choices conditioned by your worry about your health status? Is the taste of food more important than the quality when you evaluate food? Are you willing to spend more money to have healthier food? Does the thought about food worry you for more than three hours a day? Do you allow yourself any eating transgressions? Do you think your mood affects your eating behavior? Do you think that the conviction to eat only healthy food increases self-esteem? Do you think that eating healthy food changes your lifestyle (frequency of going out, friends, etc.)? Do you think that eating healthy food takes up a lot of your time? Do you feel guilty when transgressing? Do you feel satisfied with your eating behavior? Do you eat healthily? The responses were made on a four-point scale ranging from ‘never’ to ‘always’. Questions answered with ‘never’ were assigned a score of one point, while ‘always’ was assigned a score of four points. A score below thirty-five points suggests the presence of orthorexia symptoms. The Polish adaptation of the questionnaire by Stochel, Janas-Kozik et al. was developed in 2015, and the cut-off is 35 points. This means that a lower score may indicate the presence of orthorexia in the subject [37,38].

The following section uses Part A of the 2016 Polish adaptation of the Eating Attitude Test (EAT-26) questionnaire by Rogoz, Brytek-Matera, and Garner. This questionnaire assesses abnormal eating habits and the prevalence of weight concerns. Attitudes that are typical symptoms of eating disorders are examined. The EAT-26 screening test in Part A contains twenty-six questions scored on a six-point scale ranging from ‘never’ to ‘always’. This study used the following responses from the EAT26 questionnaire: I have gone on eating binges where I feel that I may not be able to stop. I cut my food into small pieces. I am aware of the calorie content of foods that I eat. I particularly avoid food with a high carbohydrate content (i.e., bread, rice, potatoes, etc.). I feel that others would prefer if I ate more. I vomit after I have eaten. I feel extremely guilty after eating. I am preoccupied with a desire to be thinner. I think about burning up calories when I exercise. Other people think that I am too thin. I am preoccupied with the thought of having fat on my body. I take longer than others to eat my meals. I avoid foods with sugar in them. I eat diet foods. I feel that food controls my life. I display self-control around food. I feel that others pressure me to eat. I give too much time and thought to food. I feel uncomfortable after eating sweets. I engage in dieting behavior. I like my stomach to be empty. I have the impulse to vomit after meals. I enjoy trying rich new foods. The questions in this section are divided into three groups: weight loss, bulimia eating control, and oral/oral control. A score of more than twenty is considered a possible eating disorder [37].

The last section of the questionnaire, designed to assess eating behaviors, is based on the TFEQ-13 (Three-Factor Eating Questionnaire). The Polish adaptation was made by Dzielska, Mazur, Małkowska-Szkutnik, and Kolo in 2009. The questionnaire consists of thirteen questions, which are answered using a four-point scale scored from zero to three points, where ‘definitely no’ equals zero points and ‘definitely yes’ equals three points. The final question is presented using an eight-point scale, where one means ‘I do not restrict at all’ and eight means ‘I always restrict’. Answers one and two are given zero points, three and four are given one point, and five and six are given two points, while answers seven and eight are assigned three points. The questionnaire is divided into three subscales. The first examines behaviours associated with food restriction in the context of type or quantity. The next subscale measures the propensity to overeat due to loss of control. The last subscale examines episodes of excessive food consumption associated with reduced mood. This study used the following responses from the TFEQ-13 and the inclusion of subscales: Cognitive Restraint: I deliberately take small helpings as a means of controlling my weight. I consciously hold back at meals in order not to gain weight. I do not eat some foods because they make me fat. Uncontrolled Eating: When I see a real delicacy, I often get so hungry that I have to eat right away. Sometimes when I start eating, I just can’t seem to stop. Being with someone who is eating often makes me hungry enough to eat also. When I see others eating, I get hungry and want to eat also. I often feel so hungry that I have to eat something right away. I can’t stop eating when I’m hungry. I get so hungry that my stomach often seems like a bottomless pit. Emotional Eating: When I feel lonely, I console myself by eating. When I feel blue, I often overeat. When I feel anxious, I find myself eating. Scores are calculated separately for each subscale. The higher the score on a subscale, the greater the severity of the disorder associated with the subscale is likely to be [39].

### 2.3. Statistical Analysis

The collected data were processed in Microsoft Office Excel 2019, while a statistical analysis was performed using the Statistica 13.3 statistical software. Statistical significance was determined using a *p*-value of 0.05. The Shapiro–Wilk test of normality was used to assess normal distributions. Descriptive statistics were performed to present data on mean and median values. The Mann–Whitney U test was used to compare data on respondents’ ages due to the presence of a non-normal distribution.

Chi-2 tests of independence were performed to examine the effect of dietary intake on individual variables, such as BMI norms, type of diet used, restriction of intake of selected products or groups of products, consumption of meat or milk substitutes, and frequency of consumption of selected food groups.

Due to the normal distribution, Student’s *t*-test was used to compare data from the ORTO-15 questionnaire results. Subsequently, the Chi-2 test of independence was used to examine the correlation between diet and the risk of orthorexia among the subjects.

Data on the median values of the EAT-26 questionnaire scores in the two study groups were compared using the Mann–Whitney U test, as the variables did not come from a normal distribution. In addition, the correlation between diet and risk of eating disorder symptoms was analysed using the Chi-2 test of independence.

Due to the non-normal distribution of the analysed variables, the median values of the scores obtained with the TFEQ-13 questionnaire among respondents from both groups were analysed using the Mann–Whitney U test.

In this study, confidence intervals (CI) were calculated to assess the precision of the estimates of the mean scores and to compare groups following different diets. Confidence intervals were set at the 95% confidence level, meaning that it can be concluded with 95% confidence that the actual mean value is within the calculated range.

## 3. Results

### 3.1. The Characteristics of the Study Group

The study included 186 people aged eighteen or over. The study group consisted of 84 people declaring that they follow vegetarian diets. In comparison, 102 people were assigned to the control group, of which 52 people (26.9%) declared that they do not follow any vegetarian diet, and 50 people (28.0%) declared that they limit meat or meat and zoonotic products. They did not consider this to be following any variation of a vegetarian diet (Table 1).

Considering the type of plant-based diet followed, an important aspect is the length of time it has been followed. In the study group (vegetarians and vegans), almost half of the respondents (47.6%) declared that they had been following a plant-based diet for over five years. Those following a vegetarian diet for one year or less were in a distinct minority (10 people in total—12.0%)—Table 2. Table 3 shows the reasons for limiting or altogether abandoning the consumption of meat and zoonotic products declared by the respondents (*n* = 186). The respondents following vegetarian diets (*n* = 84) cited ethical and environmental reasons as the main reasons for giving up meat in 86.9% of cases compared to 20.6% in the traditional diet group (*n* = 102) (*p* < 0.0001). None of those on vegetarian diets indicated prevailing fashion and dietary trends as a reason for giving up meat compared to 2.9% of those on a traditional diet (*p* = 0.3174). The influence of the environment was declared by 6.0% of vegetarians and 2.0% of those on a traditional diet (*p* = 0.3000). The belief that vegetarian diets positively affect health was an essential reason for 32.1% of vegetarians compared to 11.8% of those on a traditional diet (*p* = 0.0007). The desire to take care of their health was indicated by 42.9% of vegetarians and 29.4% of those on a traditional diet (*p* = 0.0565). Health status as a reason for giving up meat was indicated by 8.3% of vegetarians and 9.8% of those on a traditional diet (*p* = 0.7283). Aversion to the taste of meat and animal products was declared by 64.3% of vegetarians and 31.4% of those on a traditional diet (*p* < 0.0001). No vegetarians followed traditional diets and restricted their consumption of animal products compared to 43.1% of those on traditional diets (*p* < 0.0001).

### 3.2. Declared Reasons and Daily Restrictions for a Vegetarian Diet

In a survey with 84 people following vegetarian diets, 13.09% of the respondents never felt restricted in restaurants/cafes, 61.90% only experienced this sometimes, 20.23% experienced this often, and 4.76% only visited establishments with vegetarian/vegan menus. Regarding avoiding going out to restaurants/cafes because of diet, 53.57% had no such concerns, 27.38% felt this sometimes, 3.57% felt this often, and 15.47% felt no restrictions. Feeling excluded among friends and family was rarely felt by 47.61%, sometimes by 45.23%, and often by 7.14% of the respondents. Avoiding meetings due to diet was not a problem for 77.38%, sometimes a problem for 3.57%, often for 1.19%, and 17.85% did not feel restricted. When going on holiday, 44.04% of respondents did not feel excluded, 44.04% sometimes, and 11.90% often. Grocery shopping was not problematic for 58.33% of respondents, 34.52% felt constrained sometimes, and 7.14% often (Table 4).

### 3.3. An Analysis of the Results Obtained Using the ORTO-15 Questionnaire 

This study included the standardised ORTO-15 questionnaire to explore the risk of orthorexia. The lowest possible score was 15, while the highest was 60. The mean number of points obtained among the vegetarians and vegans surveyed was 35.76. In the control group, the mean score was 37.24 points. Statistically significant differences were found in the mean scores of the ORTO-15 questionnaire (*p* = 0.005) (Table 5).

**Figure 1:**
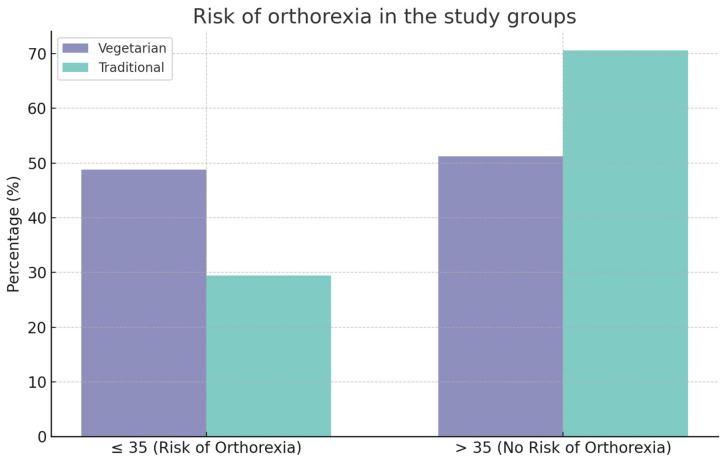
The risk of orthorexia in the study groups [*n* = 186 (100%)].

The number of scores obtained by all respondents was also analysed. A score below or equal to 35 was indicative of a risk of orthorexia. In the study group, 48.8 per cent of respondents were at risk of orthorexia, while 29.4 per cent of respondents in the control group were at risk. A significant correlation was observed between the diet followed and the risk of orthorexia symptoms. In a significantly higher proportion of subjects following a vegetarian diet, the result indicated the presence of orthorexia (48.8% vs. 29.4%; *p* = 0.00673) (Table 6 and Figure 1)

### 3.4. An Analysis of the Results Obtained Using the EAT-26 Questionnaire

Another questionnaire analysed was the EAT-26 questionnaire with 26 questions. The median total score in the group of vegetarians and vegans was 9.50 points, while it was 8.00 points in the group of those following a traditional diet. No statistically significant differences were observed between the diet followed and the median scores obtained by the respondents (*p* = 0.21762) (Table 7).

The EAT-26 questionnaire was also analysed in terms of three subscales: weight loss, bulimia and eating control, and oral control. The highest median score was the weight loss subscale, with 5.00 points in the study group and 5.00 points in the control group. The subscale with the lowest score was Subscale 3 (oral control). Significant differences were found between the midpoint values of the analysed subscale on bulimia and eating control (*p* = 0.04428). For the ‘bulimia and eating control’ subscale in the traditional group, the lack of variability means that all respondents scored the same, making it impossible to calculate the standard deviation and confidence interval. The lack of variability in responses can be a significant result (Table 8).

**Figure 2:**
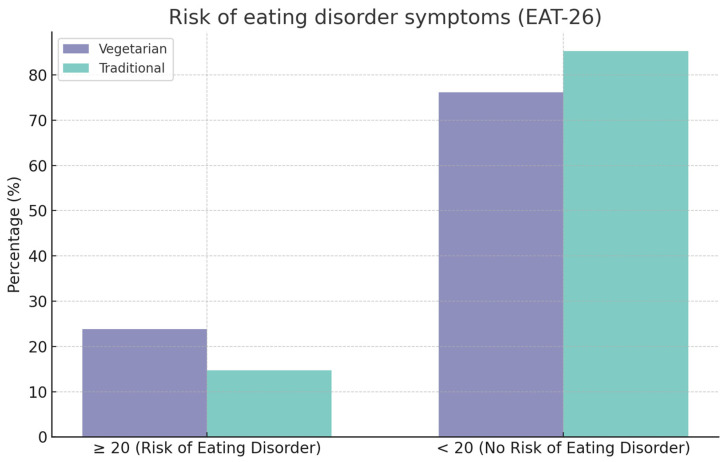
Prevalence of eating disorder symptoms among respondents [*n* = 186 (100%)] according to EAT-26 questionnaire.

The overall scores of the respondents from both groups were analysed. For 23.8% of the respondents in the vegetarian and vegan group, the overall score indicated the possibility of eating disorder symptoms. In the group of people following a traditional diet, a score indicating the existence of eating disorder symptoms was observed in 14.7%. There was no significant correlation between the diet followed and the risk of eating disorder symptoms among the respondents (*p* = 0.11391) (Table 9 and Figure 2).

### 3.5. An Analysis of the Results Obtained Using the TFEQ-13 Questionnaire

**Figure 3:**
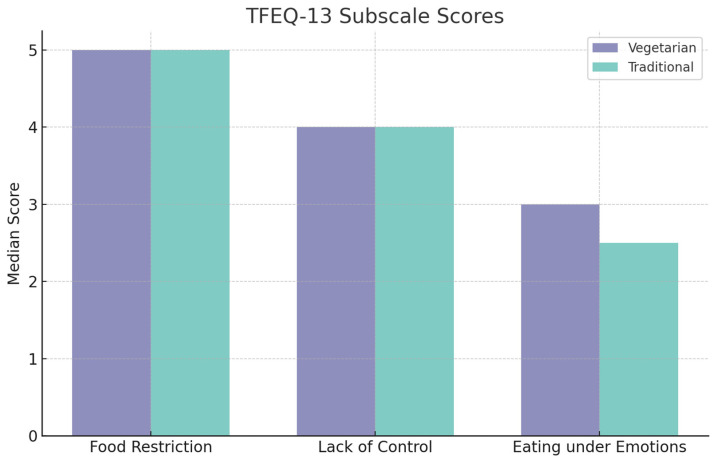
Data on TFEQ-13 questionnaire scores based on subscale in study groups [*n* = 186].

The last of the selected questionnaires was the Tri-factor Eating Questionnaire (TFEQ-13) consisting of three subscales—restricting eating, lack of control in overeating, and eating under the influence of emotions. The score of each subscale was analysed separately. The highest scores were obtained for the subscale on food restriction (M = 5.00 in both groups). No statistically significant differences existed between the study group and the control group in any of the subscales (Table 10 and Figure 3).

## 4. Discussion

It is estimated that the prevalence of vegetarianism and veganism in Europe, the United States, and Canada is between 1 and 10 per cent [40]. In 2014, according to a CBOS survey, only 1 per cent of Polish people declared themselves as vegetarians [41]. However, there are no up-to-date and reliable nationwide surveys indicating the current number of vegetarians. Data from the nationwide WOBASZ II survey (2013–2014) showed that omnivores accounted for 92.4 per cent, flexitarians for 7.4 per cent, and vegetarians for 0.16 per cent; the trends in changing eating habits have, however, changed over these ten years in Poland [42]. The prevalence of vegetarians in European studies varies from 0.5 to 4% [43,44,45,46]. Deliens et al. (2022) showed that vegetarians and flexitarians represented 1.4% and 9.2% of Flemish adults, respectively [46]. Other studies indicate that in the Swiss urban population, approximately 1.2% of the sample were vegetarians, while 15.6% declared following a flexitarian dietary pattern [47]. A high proportion of vegetarians and vegans was found in the French NutriNet-Santé study (over 3.4%) [45]. An apparent shift from an omnivorous diet towards a flexitarian diet has been noted in some societies in recent years [43,45]. These trends are likely due to public health campaigns and expert recommendations that raise awareness of the benefits and sustainability of switching to a plant-based diet. Like other middle- and high-income countries, Poland has a long tradition of eating a diet rich in animal products.

A vegetarian diet, if not properly balanced, can lead to nutritional deficiencies and malnutrition. An analysis of the relationship between diet and BMI showed no significant differences in the body mass index between the groups. In both groups, most subjects had a normal BMI (18.5–24.9 kg/m^2^). However, a study by Chiu et al. showed that the BMI of women not following vegetarian diets was higher than that of vegetarian women [48]. The differences in results may be due to the diversity of the subjects’ backgrounds. A study by Kahleov et al. showed that after 16 weeks, those on a plant-based diet reduced their BMI by approximately 2.0 kg/m^2^. This study concludes that a higher intake of plant protein may positively affect fat mass reduction [49].

There are several varieties of vegetarian diets. This study observed that the most common diet was a general vegetarian diet without a specific type. Goluch-Koniuszy et al. investigated previous varieties of vegetarianism in people who switched to a vegan diet. Lacto vegetarianism, including milk, dairy, and eggs, was most commonly declared, with 70% female and 78% male respondents [9].

An essential piece of information about vegetarian diets is their duration. The most numerous groups of vegetarians and vegans (almost half of the respondents in the study group) were characterised by more than five years of adherence to a vegetarian or vegan dietary model. Most vegans surveyed by Goluch-Koniuszy et al. (61.0% of women and 71.0% of men) declared that they had been following this diet for two to five years [9].

The main reasons for limiting animal products depend on views and values. This study found that ethical and environmental reasons were the main reasons indicated by almost 90% of vegetarians and vegans. A study by Mloda-Brylewska et al. also found that ethical considerations dominated among vegetarians (28% of women and 36% of men), and the desire to improve health was the second most common reason (22% of women and 28% of men) [50]. In the case of the self-reported survey, the desire to take care of one’s health was also the second most frequently chosen answer. The study by Sliwinska et al. also shows that as the most common reason for following a vegetarian or vegan lifestyle, the respondents choose animal protection, concern for their health, and caring for the environment—represented by 96.0%, 95.0%, and 74.0% of vegetarians surveyed, respectively [10]. In conclusion, the increasing interest in vegetarian and vegan diets highlights the necessity for further research and educational initiatives to ensure these diets are properly balanced and provide their intended health benefits. Additionally, understanding the motivations and diverse approaches to plant-based diets can aid in better aligning public health programmes with societal needs.

Orthorexia is a non-specific eating disorder that is associated with an excessive preoccupation with healthy food issues and high food restrictions [51].

Vegetarianism may influence orthorexia nervosa and other eating disorders in several ways. Research suggests that the restrictive nature of vegetarian and vegan diets may increase the risk of developing orthorexia, as individuals may become overly focused on the quality and purity of the foods they eat [52,53]. This obsession may lead to an excessive preoccupation with healthy eating that develops into an eating disorder.

Additionally, the motivations behind adopting a vegetarian diet, such as health concerns or ethical considerations, can sometimes mask existing eating disorders [54]. Studies indicate that vegetarians and vegans may have higher rates of orthorexia compared to omnivores, which is attributed to the restrictive eating patterns inherent in plant-based diets that may overlap with orthorexic behaviour [54].

In order to assess the risk of orthorexia, the validated ORTO-15 questionnaire was used in our study. The data obtained indicate that a score below 35, which represents the risk of orthorexia, was significantly more frequent in the group of people following vegetarian diets. Almost half of the vegetarians and vegans surveyed were at risk of orthorexia. A study that provides similar results was conducted by Reynolds et al. This work aimed to compare orthorexic tendencies between vegans, vegetarians, and omnivores of both the male and female sexes. It was found that just over 30.0% of the vegetarians and vegans surveyed had a score indicative of orthorexia risk. In the case of both our study and Reynolds et al.’s study, the omnivorous group had a lower risk of orthorexia with rates of 29.4% and 11.8%, respectively [55]. Another study that demonstrated a higher risk of orthorexia among vegetarians and vegans is that of Dell’Osso et al., who analysed the presence of orthorexia in Italian students. The statistical tests performed showed that vegetarians and vegans had a significantly higher prevalence of orthorexia (56.3%) [56]. In each of the studies cited, the cut-off point for the ORTO-15 questionnaire was 35 points.

Similarities between vegetarianism and psychological orthorexia include the choice of food according to specific dietary rules and the focus on food-related issues becoming a significant part of daily life [34]. However, it is not clear whether people on a vegan diet reach the cut-off threshold for pathological orthorexic behaviour. Some studies have shown that vegetarians and vegans are not only more likely to exhibit orthorexic behaviour compared to meat eaters but may also reach an initial cut-off threshold for psychological orthorexia, with vegans being more likely to exhibit these behaviours [53,57]. McLean’s narrative review showed that the ORTO 15 does not detect true pathological orthorectic eating behaviour but rather normal dietary adherence in this population [54].

In the ORTO 15, the question “Do you feel confused when you go to the grocery shop?” is included because ‘Vegans can often feel confused when reading the ingredients list and assessing whether a product contains animal products’. Conversely, question 8, “do you allow yourself to indulge in any food-related transgressions?” is included because ‘Vegans do not allow themselves to consume animal products that go beyond the limits’. These are examples of potentially misleading items in eating disorder scales for vegans such as the ORTO 15 [54].

The EAT-26 questionnaire, comprising 26 questions assessed using a six-point scale, assesses the risk of eating disorder symptoms. The existence of a risk for eating disorder symptoms is indicated by a score greater than or equal to 20. When the effect of diet on the risk of eating disorder symptoms was analysed, the presence of significant differences in the frequency of obtaining a score indicating the risk of ED (*Eating Disorder*) symptoms between the vegetarian diet group and the traditional diet group was not demonstrated. However, the study by McLean et al. indicated the presence of a statistically significant correlation between vegetarian diets and the EAT-26 questionnaire score, suggesting the presence of eating disorder symptoms, with 39 vegetarians and 101 vegans vs. 35 omnivores scoring greater than or equal to 20 points [58]. A systematic review by Sergentanis et al. noted that several studies also failed to observe a correlation between vegetarian diets and eating disorders. Nevertheless, there is also evidence that indicates an increased risk of eating disorders or the existence of a history of eating disorders among vegetarians [59].

In the Parra Fernandez study, more people following a vegan diet were at risk of developing ON (the study found that 58.2% of vegans, 24.1% of vegetarians, and 17.7% of omnivores were at risk of ON). In addition, the attitudes of vegans and vegetarians towards food were associated with ON behaviours, with the primary motivation for adopting this type of diet being the natural and healthy content of the food consumed [60].

Brytek-Matera’s results indicate that a vegetarian diet is directly related to higher levels of all aspects of orthorexia (healthy eating knowledge, healthy eating problems, and positive attitudes towards healthy eating). These findings confirm our study and the results obtained in other studies [2,61,62,63,64].

Vegetarianism is associated with greater health consciousness, including less frequent alcohol consumption, more frequent exercise, and higher fruit and vegetable consumption. This may result in higher rates of healthy orthorexia rather than psychological orthorexia. Diagnostic tools often do not distinguish between the two. Future research should focus on the multidimensional measures of orthorexia to more accurately account for psychopathology in study populations, such as vegans. Until clear and widely accepted diagnostic criteria are established, true orthorexia remains challenging to define precisely [54,57,65,66,67,68].

The increasing popularity of vegetarian and vegan diets in Europe, the United States, and Canada reflects a growing health and environmental awareness in societies. Although there is a lack of up-to-date data in Poland, changing eating habits can be observed, which are probably due to public health campaigns and recommendations from specialists. The growing interest in these diets highlights the need for further research and educational activities to help balance them properly to avoid potential nutritional deficiencies and health problems.

Vegetarianism and veganism may increase the risk of ON due to the restrictive nature of these diets and the excessive focus on the quality and purity of the foods consumed. Health and ethical motivations can sometimes mask existing eating disorders, highlighting the need for careful monitoring and support for those following these diets.

Future actions should include ensuring access to reliable information on balancing vegetarian and vegan diets, supporting and monitoring programmes to avoid nutritional deficiencies, continuing research into the health effects of plant-based diets and the relationship between vegetarian diets and orthorexia, and developing better diagnostic tools for orthorexia that take into account the specificity of plant-based diets.

## 5. The Strengths and Limitations of This Study

The limitation of this study was the size of the group. A difficulty in surveying a more significant number of people was the lack of openness of those following vegetarian diets to participate in this study. The limited size of the study group may have affected the precision and representativeness of the results. This is particularly important when analysing smaller subgroups, where the results may reflect a partial range of phenomena in the population. A larger sample size is recommended for future studies to improve the results’ reliability and generalisability. Using a recruitment method based on the snowball effect may lead to sample bias, as the people recruited by participants may have similar characteristics. This could have affected the results obtained and their representativeness.

However, this study is an innovative one. During the literature search in scientific databases, no studies dealing with the psychosocial aspects of vegetarian diets were found. In most of the papers, the reasons for following vegetarian diets were described in a very concise manner. Furthermore, the specificity of this study also relates to the selection of study groups—a group of vegetarians and vegans was compared with a group of people following traditional diets. Another strength of this study is the in-depth analysis of aspects such as the feeling of restriction in restaurants, among loved ones, or during grocery shopping or holiday trips. However, the current literature needs to provide data on this topic that can be compared with the results obtained. An essential aspect of this study is also the inclusion of questionnaires, such as the ORTO-15, which investigates the risk of orthorexia, and the EAT-26 and TFEQ-13, which provide information on the risk of eating disorder symptoms; including these items in this study resulted in the provision of many original findings, which are worth extending in the future. Three validated questionnaires (the ORTO-15, EAT-26, and TFEQ-13) allow for a comprehensive assessment of the respondents’ eating and psychosocial behaviours. Each questionnaire measures a different aspect, increasing the results’ accuracy and reliability. This study compared two groups: vegetarian diets and traditional diets. This comparison made it possible to identify differences and similarities between these groups, which is valuable for understanding the specific challenges and benefits of vegetarian diets. At the same time, the assignment to a specific group was made after analysing the data obtained in the questionnaire and not the respondents’ self-assessments.

Future research should focus on studies in the long term (so that changes can be tracked over time, which would allow for a better understanding of the long-term effects of vegetarian diets on mental health; these studies could assess how eating and health behaviours change in the long term). Future research can also include interventions aimed at reducing orthorexia in a group of people following different varieties of vegetarian diets through the use of nutritional education and working with a psychotherapist. Developing and evaluating the effectiveness of interventions to prevent and treat orthorexia among vegetarians can provide valuable data for nutritionists, physicians, and psychologists.

## 6. Practical Implications

The findings of this study have important public health, nutrition education, and psychosocial support implications for people following vegetarian diets. Learning about the main reasons for following vegetarian diets, such as ethical, health, and environmental considerations, can help in the development of educational programmes to promote healthy eating habits and balanced menus to avoid nutritional deficiencies.

The findings suggesting a higher risk of orthorexia among vegetarians and vegans highlight the need to implement psychological support programmes. Dietitians and mental health professionals should be aware of the risk of orthorexia in this group in order to offer appropriate support.

The results show that vegetarians often feel restricted in their daily lives, especially in restaurants and when shopping, suggesting the need to improve the availability and variety of plant-based options in public places, including universal access to vegan food in grocery shops or restaurants.

This study also highlights the need for further research into the psychosocial aspects of vegetarian diets, particularly in the context of eating disorder risk, to gain a more complete picture of the impact of plant-based diets on mental and social health.

## 7. Conclusions

This study found that ethical and environmental considerations were the main reasons for following vegetarian diets as opposed to people on traditional diets. People on vegetarian diets were more likely to indicate a belief in the positive effects on health and discouragement of the taste of meat. In the BMI analysis, most respondents were of average weight, but no significant differences were observed between groups. The results of the ORTO-15 questionnaire suggest that people following vegetarian diets have a higher risk of orthorexia. The EAT-26 questionnaire showed that vegetarians and vegans had a higher risk of eating disorders than those on a traditional diet. However, the differences were not statistically significant. Regarding the TFEQ-13 questionnaire, no significant differences were found between the groups for restrictive eating, lack of control in overeating, and eating under the influence of emotions. Vegetarians were more likely to experience diet-related restrictions in their daily lives. Respondents following vegetarian diets often felt restricted in restaurants and when shopping. Feelings of exclusion among friends and family were less frequently felt, and avoiding meetings for this reason was rarely declared.

## Figures and Tables

**Table 1:** The characteristics of the study group [*n* = 186].

Metric Data		People Following a Vegetarian Diet, *n* = 84 (100%)		People Following a Traditional Diet, *n* = 102 (100%)	
		*n*	%	*n*	%
Gender	Woman	82	97.6	93	91.2
	Male	2	2.4	9	8.8
Age	18–26 years	61	72.6	75	73.5
	27–40 years	16	19.0	17	16.3
	41 years and over	7	8.3	10	9.8
Professional activity	Pupil/student	29	34.5	39	38.2
	Working pupil/student	31	36.9	40	39.2
	Working person	21	25.0	21	20.6
	Unemployed person	2	2.4	1	1.0
	Other	1	1.2	1	1.0
Education	Primary school	3	3.6	0	0.0
	Gymnasium	0	0.0	3	2.9
	Vocational school	1	1.2	1	1.0
	High school	41	48.8	42	41.2
	University	39	46.4	56	54.9

**Table 2:** BMI value among respondents [*n* = 186 (100%)].

BMI	People Following a Vegetarian Diet, *n* = 84		People Following a Traditional Diet, *n* = 102		*p*-Value
	n	%	n	%	
Underweight	13	15.5	16	15.7	*p* = 0.47
Normal	58	69.0	61	59.8	
Overweight	9	10.7	18	17.6	
Obesity	4	4.8	7	6.9	

**Table 3:** Reasons for limiting or completely giving up eating meat and/or animal products as declared by respondents [*n* = 186 (100%)].

Reasons for Limiting or Completely Giving Up Eating Meat and/or Animal Products (Multiple Choice Question)	People Following a Vegetarian Diet, *n* = 84		People Following a Traditional Diet, *n* = 102		*p*-Value 0.05
	n	%	n	%	
Ethical and/or environmental considerations	73	86.9	21	20.6	<0.0001
Reigning ‘fashion’ and dietary trends	0	0.0	3	2.9	0.3174
Influence of environment (relatives/family)	5	6.0	2	2.0	0.3000
The belief that vegetarian diets have a positive impact on health	27	32.1	12	11.8	0.0007
Willingness to ‘take care’ of your health	36	42.9	30	29.4	0.0565
Health status	7	8.3	10	9.8	0.7283
Aversion to the taste of meat and/or animal products (milk and milk products. eggs)	54	64.3	32	31.4	<0.0001
I do not follow any vegetarian diet and I do not restrict my intake of animal products	0	0.0	44	43.1	<0.0001

**Table 4:** Constraints experienced by vegetarians in daily life; *n* = 84.

	People Following a Vegetarian Diet, *n* = 84	
	*n*	%
Perception of restriction in restaurants/cafes due to vegetarian diet		
Not ever	11	13.09
Only sometimes	52	61.90
So often	17	20.23
I only go to restaurants/cafes with vegetarian/vegan menus	4	4.76
Avoiding going out to restaurants/cafes due to feeling excluded because of their vegetarian diet		
Not	45	53.57
Sometimes	23	27.38
Yes	3	3.57
I don’t feel restricted	13	15.47
Perception of exclusion among friends and/or family due to vegetarian diet		
Not	40	47.61
Only sometimes	38	45.23
Yes	6	7.14
Avoiding gatherings with friends and/or family due to feelings of exclusion because of their vegetarian diet		
Not	65	77.38
Sometimes	3	3.57
Yes	1	1.19
I don’t feel restricted	15	17.85
Sense of exclusion during trips, e.g., holidays, due to vegetarian diet		
Not	37	44.04
Only sometimes	37	44.04
yes	10	11.90
Feelings of restriction when grocery shopping due to vegetarian diet		
Not	49	58.33
Only sometimes	29	34.52
yes	6	7.14

**Table 5:** Data on the results of the ORTO-15 questionnaire in the study groups [*n* = 186 (100%)].

Variable	Average	Std. Deviation	Minimum	Maximum	CI	*p*-Value	
ORTO-15 in the group of people following a vegetarian diet, *n* = 84	35.76	3.50	28.00	46.00	(34.99, 36.52)	*p* = 0.005	
ORTO-15 in the group of people following a traditional diet, *n* = 102	37.24	3.54	25.00	46.00	(36.55, 37.93)		

Student’s *t*-test was used.

**Table 6:** The risk of orthorexia in the study groups [*n* = 186 (100%)].

Risk of Orthorexia	People Following a Vegetarian Diet, *n* = 84		People Following a Traditional Diet, *n* = 102		*p*-Value
	n	%	n	%	
There is a risk of orthorexia	41	48.8	30	29.4	*p* = 0.00673
The risk of orthorexia is lower	43	51.2	72	70.6	

**Table 7:** Data on the overall scores of the EAT-26 questionnaire in the study groups [*n* = 186].

Variable	Median	Quartile Range	Minimum	Maximum	CI	*p*-Value
EAT-26 in the vegetarian diet group, *n* = 84	9.50	12.00	1.00	67.00	(7.57, 11.43)	*p* = 0.21762
EAT-26 in the group of people following a traditional diet, *n* = 102	8.00	8.00	1.00	65.00	(6.83, 9.17)	

The Mann–Whitney U test was used.

**Table 8:** EAT-26 questionnaire score data based on subscale in study groups [*n* = 186].

Questionnaire EAT-26	People Following a Vegetarian Diet, *n* = 84		People Following a Traditional Diet, *n* = 102		CI	*p*-Value 0.05
	Median	Quartile Range	Median	Quartile Range		
Subscale 1: weight loss	5.00	10.00	5.00	8.00	V (3.39, 6.61)	0.31983
					O (3.83, 6.17)	
Subscale 2: bulimia and eating control	3.00	2.00	3.00	0.00	V (2.68, 3.32)	0.04428
					O No	
					V (−0.48, 0.48)	
Subscale 3: oral control	0.00	3.00	0.00	3.00	O (−0.44, 0.44)	0.74261

The Mann–Whitney U test was used. V—vegetarians; O—omnivores.

**Table 9:** Prevalence of eating disorder symptoms among respondents [*n* = 186 (100%)] according to EAT-26 questionnaire.

Risk of Developing Eating Disorder Symptoms	People Following a Vegetarian Diet, *n* = 84		People Following a Traditional Diet, *n* = 102		*p*-Value
	n	%	n	%	
There is a risk of eating disorder symptoms	20	23.8	15	14.7	*p* = 0.11391
The risk of developing eating disorder symptoms is lower	64	76.2	87	85.3	

**Table 10:** Data on TFEQ-13 questionnaire scores based on subscale in study groups [*n* = 186].

TFEQ-13 Questionnaire	People Following a Vegetarian Diet, *n* = 84		People Following a Traditional Diet, *n* = 102		CI	*p*-Value 0.05
	Median	Quartile Range	Median	Quartile Range		
Subscale 1: food restriction	5.00	7.00	5.00	5.00	V (3.87, 6.13)	0.77279
					O (4.27, 5.73)	
Subscale 2: lack of control in overeating	4.00	4.50	4.00	5.00	V (3.28, 4.72)	0.91935
					O (3.27, 4.73)	
Subscale 3: eating under the influence of emotions	3.00	5.00	2.50	3.00	V (2.20, 3.80)	0.16612
					O (2.06, 2.94)	

The Mann–Whitney U test was used. V—vegetarians; O—omnivores.

## Data Availability

The data presented in this study are available on request from the corresponding author. The data are not publicly available due to restrictions that apply to the availability of these data.
